# Stem cell in alternative treatments for brain tumors: potential for gene delivery

**DOI:** 10.1186/2052-8426-2-24

**Published:** 2014-08-01

**Authors:** Veronica Mariotti, Steven J Greco, Ryan D Mohan, George R Nahas, Pranela Rameshwar

**Affiliations:** Department of Medicine – Hematology/Oncology, New Jersey Medical School, Rutgers School of Biomedical Sciences, E-585, 185 South Orange Avenue, Newark, NJ 07103 USA; Stowers Institute for Medical Research, Kansas City, MO USA

**Keywords:** Stem cells, Cell therapy, Glioblastoma, Cancer

## Abstract

Despite ongoing research efforts and attempts to bring new drugs into trial, the prognosis for brain tumors remains poor. Patients with the most common and lethal intracranial neoplasia, glioblastoma multiforme (GBM), have an average survival of one year with combination of surgical resection, radiotherapy and temozolomide. One of the main problems in the treatment of GBM is getting drugs across the blood brain barrier (BBB) efficiently. In an attempt to solve this problem, there are ongoing experimental and clinical trials to deliver drugs within stem cells. The purpose for this method is the ease by which stem cells home to the brain. This review discusses the experimental and clinical applications of stem cells for GBM. We also discuss the different properties of stem cells. This information is important to understand why one stem cell would be advantageous over another in cell therapy. We provide an overview of the different drug delivery methods, gene-based treatments and cancer vaccines for GBM, including the stem cell subset.

## Review

The prognosis for patients with brain tumors, in particular glioblastoma, remains poor. This poor prognosis could be explained by the hindrance to get drugs to the brain for achieving efficacious levels. Stem cells have shown tremendous promise for almost all fields of medicine including drug delivery. The application of stem cells as a cellular vehicle to deliver drugs to the brain has been noted because stem cells can cross the blood brain barrier (BBB) and can exert pathotropic effects, which attests to their ability to home to tumor sites.

Mesenchymal stem cells (MSCs) and neural stem cells (NSCs) have been used in trials to deliver prodrugs to tumors [1, 2]. However, since MSCs can also support tumor growth, this represents a major disadvantage for the application of MSCs in drug delivery to target tumors [3]. Despite this disadvantage, MSCs have a unique advantage in that they can be available upon demand, generally referred to as a source of off-the-shelf cells. This ease of availability is primarily due to their ability to evade immune rejections, thereby allowing their injection across an allogeneic barrier. It is unclear if NSCs have a similar property for use on demand or if they require matching at the major histocompatibility complex class II. This review article discusses the different issues associated with the application of stem cells as vehicles of drug delivery, using glioblastoma as a representative indication.

### Sources of stem cells - advantages/disadvantages

Embryonic (ESC), fetal and adult stem cells are intensely studied for their ability to differentiate into neuronal cells by *in vitro* methods, and are studied for their use in neurological disorders [4, 5]. Each class of stem cell possesses advantages and disadvantages for treatment of neural disorders. ESCs are derived from the inner cell mass of the blastocyst and can be differentiated *in vitro* into all cell types. Theoretically, the intrinsic ability of ESCs to form all types of neural tissues makes them superior to other stem cells. Similarly, induced pluripotent stem cells (iPSCs), which are generated through genetic manipulation of somatic cells, have the potential to form all types of cells including those within the neuronal and glial lineages [6]. The main disadvantage of ESCs and iPS is their ease in spontaneous transformation. Other issues include the ethical quandary to derive ESCs and the inefficiency to generate iPSCs.

The scientific disadvantages of ESCs and iPS led to increased interest in cell replacement strategies with adult stem cells (ASCs). More importantly, the ASCs have prospects for transplantation without ethical dilemmas. Regarding brain repair, ASCs can be effective with NSCs, MSCs, hematopoietic stem cells (HSCs) and stem cells from umbilical cord blood (UCB). Although still in experimental phase, the experimental evidence indicated that some or all of the aforementioned stem cells can differentiate into neurons and glia. There are distinct advantages of some stem cell sources over others.

NSCs are multipotent cells found within selected regions of the adult brain. NSCs can differentiate into cells of all neural lineages [7, 8]. Two neurogenic areas of the brain where NSCs reside are the subventricular zone (SVZ) of the lateral ventricles and the subgranular layer of the hippocampal dentate gyrus [9]. Physiologically, NSCs are responsible for neocortical neurogenesis to help replace damaged tissue [9]. This regenerative capacitance is outweighed by the rate of neural degeneration and the amount of damaged tissues in neurodegenerative conditions. An example of this imbalance could be seen in traumatic brain injury. Subacute NSC therapy following traumatic brain injury led to cells incorporating and remaining in the tissues two weeks after transplantation [10]. The transplanted NSCs have been shown to improve the motor function of the experimental animals [10]. A major disadvantage to the utilization of NSCs is the difficulty of harvesting and isolation from an intact brain tissue. Human NSCs can be generated from differentiated ESC, iPSCs, fetal tissues sources and cadavers. None of these sources might be able to produce adequate number of NSCs for widespread clinical implementation.

MSCs are heterogeneous, multipotent cells found in several adult tissues including bone marrow (BM) and adipose. MSCs can form cells of all germ layers [11]. In BM, MSCs are found around the central sinus where they can function as “gate-keeper” cells. At this site, the MSCs contact the abluminal region of the sinus. The presence of MSCs around the central sinus is significant to the protection of BM functions [12]. The method by which MSCs protect the BM might be important to extrapolate to other organs such as neural protection effects of MSCs.

Intravenous administration of allogeneic MSCs can promote functional recovery and brain repair in experimental ischemic stroke [13]. Due to the ease of harvesting and expanding MSCs, they can be easily available from both allogeneic and autologous sources for transplantation to patients. A major advantage of MSCs to be transplanted across allogeneic barrier makes MSCs an attractive alternative for neural repair.

BM-derived HSCs were reported to have neurogenic potential [14]. HSCs are multipotent cells with their main purpose to replenish the body’s immune and blood cells [15]. HSCs can be selected from the adult BM using well-defined markers. However, there are constraints for clinical application; in particular their low frequency in the BM, and their inability to be expanded. More importantly, there is no clear data that HSCs can generate neural cells. Overall, HSCs represent a less favorable source for neural repair.

UCB is a rich source of HSCs. The HSCs are functionally more immature as compared to similar cells in the adult BM [16]. MSCs can be isolated in the Wharton jelly of the cord and, to a lesser extent from UCB. If cord-derived MSCs can generate neurons or can be efficient in neural repair, this would be a major advantage because there is no risk to the donor since the cord would be otherwise discarded. Regarding hematopoietic replacement with UCB cells, there is a limitation because of the low volume of blood. This would provide inadequate number of HSCs for transplantation.

### Drug delivery - blood brain barrier (BBB)

The brain has limited regenerative capacity sufficient only to replace modest numbers of lost cells [17, 18]. The brain has shown a surprising ability for spontaneous repair in patients with stroke [19]. Accordingly, special biological protections exist to protect this vital organ, making the brain a difficult target for delivery of therapeutics. These include the hard barrier created by the skull, three meninges membranes of varying thickness/toughness, cerebrospinal fluid, and the BBB. The brain is well-protected by the BBB. The BBB is a biological fortress created primarily by a sheet of tightly knit endothelial cells. The cellular junctions are tight so that even small molecules have difficulty to cross the BBB. The BBB effectively seals off the brain from the rest of the body and enables strict selectivity in what crosses into the brain. In practice, this means that most therapeutics will not pass through the BBB. This can be problematic even for diseases affecting tissues other than the brain. For example, secondary tumors arising after breast cancer treatment can be found in the brain. This is thought to occur because most anticancer treatments do not pass through the BBB, leaving metastasized cells to reside in the brain. At a later date, they can emerge from quiescence to form deadly tumors [20].

Stem cells have been proposed as a cellular vehicle to deliver therapeutic agents to the brain. Due the difficulty of treating glioblastoma this type of cancer forms a basis to test if drugs delivered within stem cells can be effective in targeting the cancer cells (Table 1). There are a number of strategies by which the stem cells are delivered to facilitate passage of therapeutics through the BBB (Table 2). Intranasal delivery of stem cells is not invasive and shows promise as a method of treatment. In an experimental mouse model, drug-loaded MSCs were delivered through the intranasal route and resulted in effective treatment of glioma [21, 22]. Other varied approaches range from physical to chemical methods to evade the hurdles associated with the BBB to deliver drugs. An example of physical bypass of the BBB involved the use of surgical implantation of cells and drug-soaked discs designed to release the drugs in a time-dependent manner. This method has the advantage of allowing very specific drug dosing by placing the cells near or within the tumors or lesions. The disadvantage of this method is the disruption of BBB integrity caused by the surgery. In another physical approach, drug or stem cells are released outside the BBB while tight BBB junctions are loosened by ultrasound [23]. This has the advantage of temporarily affecting BBB integrity, but leaves the dosage to be empirically determined. The tight intercellular junction requires that molecules are modified for traversing the BBB. Since the junction heavily favors lipophilic molecules, drugs are chemically modified by lipidation and glycosylation [24]. An alternative approach is to load the drugs in liposomes for passage through the BBB [25, 26]. These strategies would allow for control of the drugs passing into the brain. However, increased amount of drugs into the brain can be hazardous because passage out of the BBB would be subjected to the same constraints as passage into the BBB. Thus, there will be an accumulation of drug metabolites that could lead to toxicity over time.

**Table 1:** **Stem cells in drug delivery to treat brain tumors**

Stem cell	Sources	Tumor type	References
**MSC**	Bone marrow	Glioblastoma	[27–31]
	Adipose	Glioblastoma	[27, 28, 30–32]
**NSC**	Fetal brain	Medulloblastoma, Glioma	[33–36]

Shown are two different types of stem cells, obtained from varied sources to treat brain tumors.

**Table 2:** **Methods to deliver stem cells or drugs to the brain**

	Method	Indication
Physical	Intranasal delivery	Cells/Drugs
	Surgical	Cells/Drugs
	Ultrasound disruption	Cells/Drugs
Chemical	Lipidation, glycosylation	Drugs
	Loading on to liposomes	Drugs

The table shows the use of specific methods to deliver cells and drugs to the brain [21, 22].

In the future, investigation into novel pathways regulating BBB permeability may lead to new strategies for modulating the permeability. There are some indications that BBB permeability is subject to alterations in some diseases. For example, neurodegenerative diseases such as neuropsychiatric systemic lupus erythematosus and Parkinson’s disease have been reported to show increased permeability of the BBB [37, 38]. The BBB can also be altered under non-disease conditions to facilitate the immune response. This could occur by the involvement of components associated with the BBB such as pericytes. The pericytes can respond to changes in the microenvironment by changing their structures and shapes to modulate BBB permeability [39]. If it were possible to mimic the pathways responsible for opening the BBB for immune response, methods could be developed to trick the BBB into temporarily opening up to allow delivery of cellular therapies.

### Viral gene therapy

**Figure 1:**
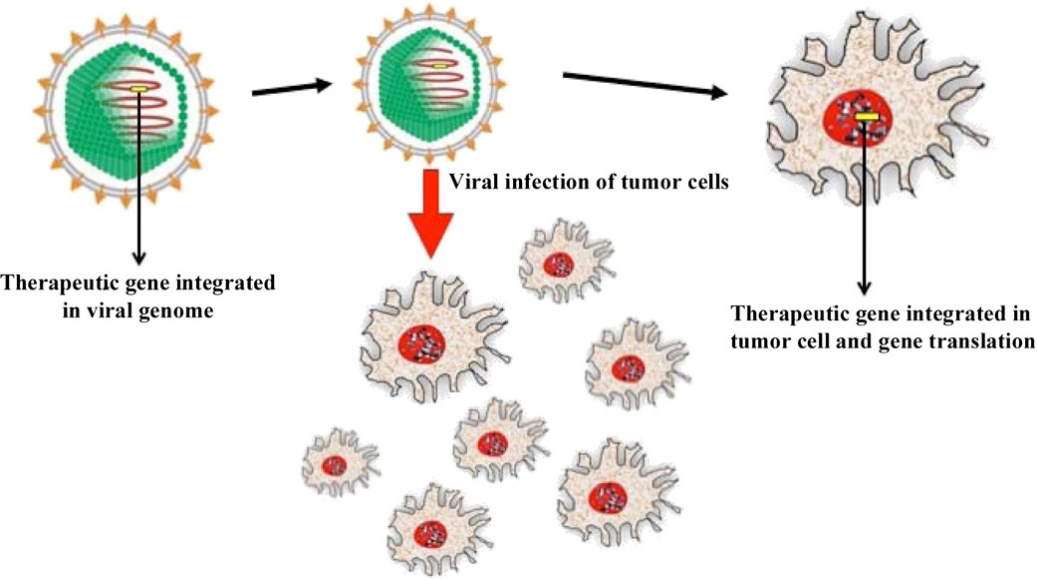
**Use of oncolytic viral transfer into cancer cells.** The figure is a schematic representation of viral transfer to target rapidly dividing cancer cells. The gene of interest is integrated in the viral genome (left). The engineered virus is administrated and infects rapidly dividing cells, like cancer cells (center). Infected cells then have integrated DNA of the gene of interest that was carried by the virus, resulting in their expression (right).

This section discusses approaches involved in the use of engineered viruses to deliver enzymes that can convert prodrugs into toxic metabolites. Viruses preferentially infect rapidly dividing tumor cells. Figure 1 shows how virus-containing genes could be packaged for cancer therapy. Here, the non-dividing nature of neural cells would be spared by the infection to deliver the virus. A commonly used method is to combine the gene delivery system with a gene that expresses the enzyme, thymidine kinase, followed by ganciclovir treatment. The infected cells are able to translate the enzyme thymidine kinase, which phosphorylates ganciclovir to ganciclovir triphosphate. As a result, only the infected, rapidly dividing cells, are killed by the toxic metabolites and apoptosis is induced both in transduced cells and also adjacent dividing cells (“bystander effect”) [40]. Numerous preclinical and phase I/II studies have investigated this procedure and showed promising results. In an i*n vivo* study using a rat model of cerebral glioma, fibroblasts carrying the herpes simplex virus type 1 thymidine kinase (HSV-tk) gene through retroviral vector were stereotactically injected and subsequently treated with ganciclovir (GCV) to produce a therapeutic response [41]. Clinically, a pilot trial using HSV-tk-containing adenoviruses (AdV-tk) and GCV in 13 patients with recurrent malignant brain tumors showed acceptable toxicity [42].

A phase Ib trial with relatively few patients (n = 12) who were newly diagnosed with malignant glioma received AdV-tk via tumor bed injection at time of surgery. This was followed by valaciclovir with overlapping radiation therapy, then treatment with the chemotherapeutic temozolomide. The treatment showed no significant toxicity and resulted in survival of 33% at 2 years and 25% at 3 years. The patient-reported quality of life was stable or improved after treatment. Significant T-cell inflammatory infiltrate was found in four re-resected tumors, implying long-lasting immune stimulation [43].

A phase II trial, which utilized an adenoviral vector to treat initial and recurrent high-grade gliomas showed a significant improvement in survival when compared with historical controls. However, the subsequent randomized phase II trials did not show any statistically significant improvement in survival [44]. Based on these conflicting results in preclinical and phase I/II studies, a phase III study was conducted in adults with previously untreated GBM [45]. This trial utilized HSV-tk and GCV gene therapy as an adjuvant to surgical resection and radiation. The trial involved 248 patients that received either surgical resection and radiotherapy, or surgical resection and radiotherapy plus adjuvant gene therapy at the time of surgery. The experimental treatment was confirmed to be safe; however no difference was noted for overall survival or disease progression.

A more recent phase III study (n = 250) on the clinical use of Adenovirus-mediated gene therapy with sitimagene ceradenovec (replication-deficient adenovirus) followed by intravenous GCV (ASPECT trial) has been conducted [46]. The study compared standard of care (surgical resection followed by radiotherapy and chemotherapy) with perilesional injection of sitimagene ceradenovec followed by GCV. The median time to death or re-intervention was longer in the experimental group (*p* = 0.006) and, in a subgroup of patients with non-methylated methylguanine-DNA methyltransferase (MGMT) the hazard ratio (HR) was 1.72 (95% CI 1.15-2.56; *p* = 0.008). However, there was no difference between the two groups in terms of overall survival. One possible explanation for the discordant results observed in the studies is the variable gene delivery and transduction rate in the tumor cells, which has been rarely measured. This observation encourages further efforts focused on the optimization of drug delivery administration [47].

### Use of stem cells in glioblastoma treatment

Neoplasias, including gliomas, are heterogeneous diseases. There is overwhelming *in vitro* and *in vivo* evidence that a subpopulation of cancer stem cells (CSCs) initiates and sustain tumor growth, resulting in tumor masses with heterogeneous malignant cells [48]. It has also been suggested that a subpopulation of glioma CSCs (gCSCs) may be responsible for the resistance to standard therapy. Eradication of the gCSCs could lead to significant improvement in patients’ outcomes [49].

### Targeting cancer stem cells (CSCs)

Targeting CSCs could be most efficient to prolong the survival of cancer patients. Several microRNAs, such as miRNA-145 and oncomiR-138, have been identified for this purpose. The oncomiR-138 is considered to be a molecular signature of gCSC and has been targeted by functional inhibition *in vitro*, resulting in decreased tumorigenesis and impairment of gCSCs growth [50]. *In vitro* studies showed a tumor suppressive effect of miRNA-145 in glioblastoma [51]. The suppressive effects of miR-145 occur by its ability to decrease the expression of stem cell-linked genes within the CD133 expressing CSC-like cells [51]. Ectopic delivery of miR-145, in combination with radiotherapy and chemotherapy, improved survival in an experimental model of glioblastoma [51]. The inhibition of the Notch pathway through gamma-secretase inhibitors has also been experimentally studied as a targeted treatment for gliomas [52]. This was performed by the implantation of a drug-impregnated, polymer bead delivery system. This treatment method blocked tumor growth and prolonged the survival of a small cohort of mice.

### Stem cells in gene and drug delivery

Studies are proposed with NSCs as a possible cellular vehicle to deliver suicide genes to tumors. The advantage of this approach is that NSCs are tumor tropic. The tropic effects are likely facilitated by the production of several chemoattractants by glioma cells, such SCF-1 or MCP-1 [53]. Tables 1 and 2 show a snapshot of how drugs can be delivered to brain tumors with the use of stem cells. Also shown in Table 2 are the different approaches to find the most efficient method to deliver the drug-loaded stem cells to the brain.

There are few reports describing the use of NSCs to deliver the enzyme cytosine deaminase (CD), followed by treatment with 5-Flucytosine (5-FC). 5-FC can be converted by CD to the cytotoxic compound 5-fluorouracil (5-FU), which selectively targets tumor cells, with minimal toxicity to the surrounding healthy tissues [1]. Clinical trials using a similar approach are ongoing [54]. Programmed self-destructive NSCs have also been used as a delivery tool for pH-sensitive Mesoporous Silica Nanoparticles-doxorubicin (MSN-dox); the NSCs migrate to the tumor site, eventually undergo apoptosis and release the MSN-dx to the surrounding gCSCs. [55]. MSC have been used in several experimental models as an efficacious tool for drug delivery. Delivery of soluble (s)-TRAIL via MSCs has been shown to induce apoptosis in glioma. Mice were implanted with a mix of gCSCs and S-TRAIL-expressing MSCs or a mix of gCSCs with GFP-expressing MSCs as control. Real time imaging showed a significant reduction in tumor burden in animals implanted with MSCs expressing s-TRAIL as compared to controls [56].

Recent studies showed a role for miR-9 in the expression of the drug efflux transporter, P-glycoprotein in TMZ-resistant GBM cells [2]. MSCs loaded with anti-miR-9 were able to deliver the drug through exosomes thereby re-sensitizing the GBM cells to TMZ [2]. MSCs were used to deliver the CD suicide gene *in vivo* [27]. Intracerebral inoculation of engineered MSCs after glioblastoma surgical resection resulted in a curative outcome in a significant number of mice [27].

Marrow-isolated adult multilineage inducible (MIAMI) cells, which are believed to be a subset of MSCs, have been used to deliver lipid nanocapsule loaded with an organometallic complex [28]. *In vitro* and *in vivo* studies indicated that this type of drug delivery resulted in cytotoxic effect on the glioma cells [28]. MSCs, engineered to express a single-chain antibody to EGFRvIII, were co-injected with EGFRvIII (+) glioblastoma in a xenograft model. The MSCs-EGFRvIII showed significant increase in the survival of the mice as compared to the controls injected with glioma cells alone [57].

### Oncolytic viruses

Another possible approach to treat brain tumors involves the use of oncolytic viruses. These viruses preserve the ability to replicate, selectively amplifying in cancer cells to cause cytotoxicity. The oncolytic virus can also activate and stimulate the natural immune response to target infected cells. One example of a widely used oncolytic virus is herpes simplex virus (HSV), primarily because of its cytotoxicity and ability to induce a strong immune response [58].

Several phase I/II studies investigated this technique using various engineered viruses, such as HSV, adenovirus, retrovirus and others, and showed overall safety, low rate of complications, and promising effects [59–61]. Cheema et al. combined oHSV and IL-12 to enhance the immune response and at the same time prevent angiogenesis [62]. The transgenic oHSV (G47Δ-mIL12) showed a significant increase in survival, decreased neovascularization, decreased VEGF expression, and increased production of angiostatic IP-10 (CXCL10). The efficacy of the transgenic oHSV was markedly reduced in athymic mice, suggesting a direct effect of T-cell stimulation with IL-12.

### Anti-cancer vaccines and stem cells

The use of anti-cancer vaccines has been studied for the treatment of several solid tumors, resulting in discordant results [63]. These therapies re-sensitize and enhance the natural immune response of the host against tumor cells. In the absence of the vaccine the tumors can evade the immune response.

One method involves the use of dendritic cells (DCs) from patients for sensitization to the tumor’s unique antigens. The DCs are then reinfused in the patient, where they can activate cytotoxic T-cells against the tumor cells. Several phase I and II studies showed feasibility and safety of this approach in the treatment of gliomas, with some encouraging results [64–66]. Another easier approach to cancer vaccine is the use of formalin fixed tumor cells, collected at tumor’s resection time, as a stimulant for immune response. This approach has been studied in phase I/II studies with promising results [67]. The combination of cancer vaccine with EGFR inhibitors has also been explored in a phase II study, showing safety and increased survival [68].

Targeting the subpopulation of glioma Cancer Stem Cells (gCSCs) through immunotherapy could lead to the eradication of gCSCs. This particular subpopulation of cells is therefore the ideal candidate for vaccine therapy. Ji et al. took advantage of CD133, a proposed marker of gCSCs, as a target to stimulate cytotoxic T-cells (CTLs) [69]. Peptide-specific CD8^+^ CTLs from normal donors were generated and pulsed with autologous DCs. The CTLs efficiently recognized the CD133 epitopes and were specifically able to lyse CD133+ gCSCs *in vivo*.

### Chimeric antigen receptor

**Figure 2:**
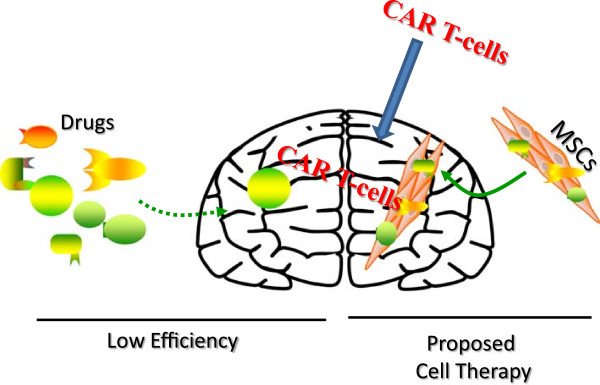
**Relative efficiency of drug delivery in cells or alone.** Left: Shows the low efficiency of drugs entering the brain due to the protection of the BBB. Right: Shows the potential to increase the efficiency of drug delivery when the drug is applied within MSCs. Center: Shown is the efficiency of CAR T-cells to enter the brain.

Chimeric antigen receptor (CAR) T-cells were first developed in an attempt to expand the therapeutic potential of effector lymphocytes in adoptive T-cell transfer, a term first coined in the 1950s [70]. In a landmark study, the single-chain of an Fv (scFv) antibody molecule was fused to the *γ* chain of the Fc receptor or to the ζ of the CD3 complex, creating T cells with antibody type specificity and subsequent IL-2 signaling leading to target cell lysis [71]. This target-binding site displays an affinity much higher than TCRs, and in addition is MHC independent, avoiding tumor escape mechanisms secondary to MHC loss variants. The advancements in the field to characterize “designer lymphocytes” provide a scaffold for cell-based immunotherapies. Here we discuss the CAR T-cells as a method of immunotherapy for brain tumors because these cells show enhanced efficiency to enter the brain (Figure 2). Indeed, there are ongoing studies to use CAR T-cells to EGFR, a common receptor on glioblastoma [72].

Over the years, CARs have been engineered and manipulated to achieve more targeted and potent effects. The need for this became clear upon further understanding of activating ligands on antigen presenting cells such as CD80 and CD86 that bind to the co-stimulatory receptors found on T-cells including, but not limited to CD28 [73]. Further focus turned towards incorporating co-stimulatory signals into the domain in order to prevent T-cell apoptosis or anergy [74, 75]. This has otherwise led to the development of second and third generation CARs with greater effects than previous generations.

The advantages of CAR therapy are many including HLA-independent recognition of target antigens and the ability to rapidly deliver a large population of tumor antigen-specific T-cells. However, there are important disadvantages that must be addressed, including 'on target/off tumor’ effects, explained by antigen similarities that may be shared by normal cells and tumor cells, and cytokine-release syndrome.

Cytokine-release syndrome is driven by pro-inflammary cytokines such as IFN-γ, TNF-α and IL-2 [76, 77] or more recently described, IL-6 [78]. The effects of IL-6 was demonstrated with studies using toclizumab (anti IL-6 receptor monoclonal antibody) in glucocorticoid resistant GvHD [79]. Cytokine-release syndrome seems to be related, however with T-cell expansion as patients present clinically with fever, variable degrees of myalgias, nausea and anorexia and with complications that ranged from hemodynamic or respiratory instability [76].

Medically, there are clinical manifestations that limit the use of CAR therapy. CAR therapy is contingent on the immunogenicity of the target cells. The growing field of cancer stem cells (CSCs) showed that they should be the target cells. The identification of markers on CSCs is a subject of investigation, lead to questions. How immunogenic are CSCs? There are also concerns to the fate of CAR cells upon entering the tumor microenvironment. Lastly, most data pertains to hematological malignancies; yet some data, although limited with modest or null results, have been done on solid tumors, including neuroblastomas [80].

There are recent advances with promising results in CAR therapy. The mechanisms behind cytokine-release syndrome and its associated neurotoxicities must be addressed. These limitations may further be explored with future studies that include greater power. This may pave the way for engineering of other cells, as CAR modifiable cells are not limited to T-cells, but also include, but are not limited to NK cells, iNKT cells.

## Conclusion

Glioblastoma’s aggressive behavior seems to be determined by the extreme heterogeneity of this tumor, which can be sustained by gCSCs. The CSCs can generate, re-generate and maintain tumor growth. The challenges related to the treatment of glioblastoma are represented in the first place by the BBB, which physically isolates the brain from the rest of the body, causing difficult delivery of chemotherapeutics and reducing the access of cells of the immune system. Figure 2 shows the relative efficiency of delivering drugs directly or through stem cells. The stem cells show promise to enter the brain with higher efficiency to directly deliver the drugs to brain.

Glioblastoma is indeed often able to evade the natural host’s immune response. Studies focusing on successful delivery of therapeutics have been conducted to determine if the permeability of BBB can be modulated to enhance the transport of drugs to the brain. Further studies focused on the regulatory pathways of the BBB’s permeability could allow delivery of targeted therapies in specific time frames.

Different types of stem cells have been studied as a method of optimal delivery of suicide genes and drugs, due to their unique ability to migrate to the tumor bed with adequate specificity (Table 3). MSCs can be relatively easily harvested and expanded, however concerns regarding the potential for transformation needs to be thoroughly addressed [81].

**Table 3:** **Cancer targeting agents delivered by stem cells for glioblastoma**

Drug/gene	Stem cell	Targeting method	References
Cytosine deaminase (CD)	NSCs	Indirect via conversion of prodrug, 5-fluorocytosine (5-FC)	[36]
CD	MSCs	Indirect, via conversion of the prodrug. 5-FC	[27]
MSN-dox	NSCs	Direct	[55]
Soluble TRAIL	MSCs	Direct	[56]
miR-9	MSCs	Direct	[2]
Fc-diOH-LNC	MIAMIs	Direct	[28]

Shown are representatives methods by which drug/gene/RNA can be used in stem cell delivery system for the treatment of glioblastoma. Direct method indicates that the stem cells release the drug, which interacts with the cancer cells for cytotoxic effects. Indirect effects are indicated when an enzyme is delivered in the stem cells for the local conversion of a prodrug to its active form.

Gene therapy has been broadly studied for glioblastoma, and various techniques for gene delivery involving the use of stem cells as transporters, have been investigated with promising results *in vivo* and in preclinical studies; unfortunately phase III clinical trials failed to demonstrate a clear advantage of experimental therapies in terms of OS. This could be explained by low delivery rate and/or inconsistent levels of transductions of the drugs, therefore further research focused on improvement of delivery methods could potentially bring significant improvements to glioblastoma gene-based treatments [47].

Finally, great hope is represented by strategies based on the enhancement of natural host immune response, which is frequently evaded by the tumor: anti-cancer vaccines targeting gCSCs would theoretically allow the eradication of this cell subpopulation, likely responsible of recurrence and resistance to chemotherapy. This study addresses the use of CAR T-cells, which shows promise. Its inclusion in this brief review is mainly due to the relative ease to enter the brain [72].

The use of MSCs or NSCs as a delivery tool for either specific genes or drugs has been explored *in vitro* and *in vivo* showing promising results in terms of tumor cells cytotoxicity and increased survival in animal models. These methods clearly offer enormous advantages for the potential treatment of brain tumors, being able of targeting almost uniquely cancer cells. Few clinical trials addressing the feasibility and safety of these approaches are ongoing (Table 2).

In conclusion, as an extreme heterogeneous disease, complicated by the peculiar localization in a selectively protected environment, glioblastoma is a disease that needs to be approached from different angles, keeping in mind the unique limits and potentials of the organ from which it raises from. These features, even though extremely challenging, offer potential starting points for future research.

## References

[1] Kim SU, Jeung EB, Kim YB, Cho MH, Choi KC (2011). Potential tumor-tropic effect of genetically engineered stem cells expressing suicide enzymes to selectively target invasive cancer in animal models. Anticancer Res.

[2] Munoz JL, Bliss SA, Greco SJ, Ramkissoon SH, Ligon KL, Rameshwar P (2013). Delivery of functional anti-miR-9 by mesenchymal stem cell-derived exosomes to glioblastoma multiforme cells conferred chemosensitivity. Mol Ther Nucleic Acids.

[3] Greco SJ, Rameshwar P (2012). Mesenchymal stem cells in drug/gene delivery: implications for cell therapy. Ther Deliv.

[4] Chang DJ, Oh SH, Lee N, Choi C, Jeon I, Kim HS, Shin DA, Lee SE, Kim D, Song J (2013). Contralaterally transplanted human embryonic stem cell-derived neural precursor cells (ENStem-A) migrate and improve brain functions in stroke-damaged rats. Exp Mol Med.

[5] Wang S, Cheng H, Dai G, Wang X, Hua R, Liu X, Wang P, Chen G, Yue W, An Y (2013). Umbilical cord mesenchymal stem cell transplantation significantly improves neurological function in patients with sequelae of traumatic brain injury. Brain Res.

[6] Takahashi K, Yamanaka S (2006). Induction of pluripotent stem cells from mouse embryonic and adult fibroblast cultures by defined factors. Cell.

[7] Emsley JG, Mitchell BD, Kempermann G, Macklis JD (2005). Adult neurogenesis and repair of the adult CNS with neural progenitors, precursors, and stem cells. Prog Neurobiol.

[8] Young HE (2004). Existence of reserve quiescent stem cells in adults, from amphibians to humans. Curr Top Microbiol Immunol.

[9] Sanai N, Alvarez-Buylla A, Berger MS (2005). Neural stem cells and the origin of gliomas. N Engl J Med.

[10] Harting MT, Sloan LE, Jimenez F, Baumgartner J, Cox CS (2009). Subacute neural stem cell therapy for traumatic brain injury. J Surg Res.

[11] Phinney DG, Prockop DJ (2007). Concise review: mesenchymal stem/multipotent stromal cells: the state of transdifferentiation and modes of tissue repairΓÇöCurrent views. Stem Cells.

[12] Castillo M, Liu K, Bonilla L, Rameshwar P (2007). The immune properties of mesenchymal stem cells. Int J Biomed Sci.

[13] Gutierrez-Fernandez M, Rodriguez-Frutos B, Ramos-Cejudo J, Teresa Vallejo-Cremades M, Fuentes B, Cerdan S, Diez-Tejedor E (2013). Effects of intravenous administration of allogenic bone marrow- and adipose tissue-derived mesenchymal stem cells on functional recovery and brain repair markers in experimental ischemic stroke. Stem Cell Res Ther.

[14] Sigurjonsson OE, Perreault MC, Egeland T, Glover JC (2005). Adult human hematopoietic stem cells produce neurons efficiently in the regenerating chicken embryo spinal cord. Proc Natl Acad Sci.

[15] Weissman IL (2000). Stem cells: units of development, units of regeneration, and units in evolution. Cell.

[16] Kogler G, Radke TF, Lefort A, Sensken S, Fischer J, Sorg RV, Wernet P (2005). Cytokine production and hematopoiesis supporting activity of cord blood-derived unrestricted somatic stem cells. Exp Hematol.

[17] Nakagomi T, Taguchi A, Fujimori Y, Saino O, Nakano-Doi A, Kubo S, Gotoh A, Soma T, Yoshikawa H, Nishizaki T, Nakagomi N, Stern DM, Matsuyama T (2009). Isolation and characterization of neural stem/progenitor cells from post-stroke cerebral cortex in mice. Eur J Neurosci.

[18] Nunes MC, Roy NS, Keyoung HM, Goodman RR, McKhann G, Jiang L, Kang J, Nedergaard M, Goldman SA (2003). Identification and isolation of multipotential neural progenitor cells from the subcortical white matter of the adult human brain. Nat Med.

[19] Cramer SC (2008). Repairing the human brain after stroke: I. Mechanisms of spontaneous recovery. Ann Neurol.

[20] Peddi PF, Hurvitz SA (2014). PI3K pathway inhibitors for the treatment of brain metastases with a focus on HER2+ breast cancer. J Neurooncol.

[21] Balyasnikova IV, Prasol MS, Ferguson SD, Han Y, Ahmed AU, Gutova M, Tobias AL, Mustafi D, Rincon E, Zhang L, Aboody KS, Lesniak MS (2014). Intranasal delivery of mesenchymal stem cells significantly extends survival of irradiated mice with experimental brain tumors. Mol Ther online.

[22] Burgess A, Ayala-Grosso CA, Ganguly M, Jordao JF, Aubert I, Hynynen K (2011). Targeted delivery of neural stem cells to the brain using MRI-guided focused ultrasound to disrupt the blood–brain barrier. PLoS One.

[23] Aryal M, Arvanitis CD, Alexander PM, McDannold N (2014). Ultrasound-mediated blood–brain barrier disruption for targeted drug delivery in the central nervous system. Adv Drug Deliv Rev.

[24] Varamini P, Toth I (2013). Lipid- and sugar-modified endomorphins: novel targets for the treatment of neuropathic pain. Frontiers Pharmacol.

[25] Bhujbal SV, de Vos P, Niclou SP (2014). Drug and cell encapsulation: alternative delivery options for the treatment of malignant brain tumors. Adv Drug Deliv Rev.

[26] Gurudevan S, Kanwar RK, Veedu RN, Sasidharan S, Kennedy RL, Walder K, Prasad N, Kanwar JR (2013). Targeted multimodal liposomes for nano-delivery and imaging: an avenger for drug resistance and cancer. Curr Gene Ther.

[27] Altaner C, Altanerova V, Cihova M, Ondicova K, Rychly B, Baciak L, Mravec B (2014). Complete regression of glioblastoma by mesenchymal stem cells mediated prodrug gene therapy simulating clinical therapeutic scenario. Int J Cancer.

[28] Roger M, Clavreul A, Huynh NT, Passirani C, Schiller P, Vessieres A, Montero-Menei C, Menei P (2012). Ferrociphenol lipid nanocapsule delivery by mesenchymal stromal cells in brain tumor therapy. Intl J Pharmaceutics.

[29] Kim SM, Woo JS, Jeong CH, Ryu CH, Jang JD, Jeun SS (2014). Potential application of temozolomide in mesenchymal stem cell-based TRAIL gene therapy against malignant glioma. Stem Cells Transl Med.

[30] Kim SM, Woo JS, Jeong CH, Ryu CH, Lim JY, Jeun SS (2012). Effective combination therapy for malignant glioma with TRAIL-secreting mesenchymal stem cells and lipoxygenase inhibitor MK886. Cancer Res.

[31] Roger M, Clavreul A, Venier-Julienne MC, Passirani C, Sindji L, Schiller P, Montero-Menei C, Menei P (2010). Mesenchymal stem cells as cellular vehicles for delivery of nanoparticles to brain tumors. Biomaterials.

[32] Choi SA, Lee JY, Wang KC, Phi JH, Song SH, Song J, Kim SK: **Human adipose tissue-derived mesenchymal stem cells: characteristics and therapeutic potential as cellular vehicles for prodrug gene therapy against brainstem gliomas.** *Eur J Cancer* **48:**129–137. 10.1016/j.ejca.2011.04.033 21664124

[33] Kim SK, Kim SU, Park IH, Bang JH, Aboody KS, Wang KC, Cho BK, Kim M, Menon LG, Black PM, Carroll RS (2006). Human neural stem cells target experimental intracranial medulloblastoma and deliver a therapeutic gene leading to tumor regression. Clin Cancer Res.

[34] Metz MZ, Gutova M, Lacey SF, Abramyants Y, Vo T, Gilchrist M, Tirughana R, Ghoda LY, Barish ME, Brown CE, Najbauer J, Potter PM, Portnow J, Synold TW, Aboody KS (2013). Neural stem cell-mediated delivery of Irinotecan-activating carboxylesterases to glioma: implications for clinical use. Stem Cells Transl Med.

[35] Ahmed AU, Thaci B, Tobias AL, Auffinger B, Zhang L, Cheng Y, Kim CK, Yunis C, Han Y, Alexiades NG, Fan X, Aboody KS, Lesniak MS (2013). A preclinical evaluation of neural stem cell-based cell carrier for targeted antiglioma oncolytic virotherapy. J Natl Cancer Inst.

[36] Aboody KS, Najbauer J, Metz MZ, D’Apuzzo M, Gutova M, Annala AJ, Synold TW, Couture LA, Blanchard S, Moats RA, Garcia E, Aramburo S, Valenzuela VV, Frank RT, Barish ME, Bown CE, Kim SU, Badie B, Portnow J (2013). Neural stem cell-mediated enzyme/prodrug therapy for glioma: preclinical studies. Sci Transl Med.

[37] Stock AD, Wen J, Putterman C (2013). Neuropsychiatric lupus, the blood brain barrier, and the TWEAK/Fn14 pathway. Front Immunol.

[38] Lee H, Pienaar IS (2014). Disruption of the blood–brain barrier in Parkinson’s disease: curse or route to a cure?. Front Biosci.

[39] Hurtado-Alvarado G, Cabanas-Morales AM, Gomez-Gonzalez B (2014). Pericytes: brain-immune interface modulators. Front Integr Neurosci.

[40] Marsh JC, Goldfarb J, Shafman TD, Diaz AZ (2013). Current status of immunotherapy and gene therapy for high-grade gliomas. Cancer Control.

[41] Culver KW, Ram Z, Wallbridge S, Ishii H, Oldfield EH, Blaese RM (1992). In vivo gene transfer with retroviral vector-producer cells for treatment of experimental brain tumors. Science.

[42] Trask TW, Trask RP, Aguilar-Cordova E, Shine HD, Wyde PR, Goodman JC, Hamilton WJ, Rojas-Martinez A, Chen SH, Woo SLC, Grossman RG (2000). Phase I study of adenoviral delivery of the HSV-tk gene and Ganciclovir administration in patients with recurrent malignant brain tumors. Mol Ther.

[43] Chiocca EA, Aguilar LK, Bell SD, Kaur B, Hardcastle J, Cavaliere R, McGregor J, Lo S, Ray-Chaudhuri A, Chakravarti A, Grecula J, Newton H, Harris KS, Grossman RG, Trask TW, Baskin DS, Monterroso C, Manzanera AG, Aguilar-Cordova E, New PZ (2011). Phase IB study of gene-mediated cytotoxic immunotherapy adjuvant to up-front surgery and intensive timing radiation for malignant glioma. J Clin Oncol.

[44] Sandmair AM, Loimas S, Puranen P, Immonen A, Kossila M, Puranen M, Hurskainen H, Tyynela K, Turunen M, Vanninen R, Lehtolainen P, Paljarvi L, Johansson R, Vapalahti M, Yla-Herttuala S (2000). Thymidine kinase gene therapy for human malignant glioma, using replication-deficient retroviruses or adenoviruses. Hum Gene Ther.

[45] Rainov NG (2000). A phase III clinical evaluation of herpes simplex virus type 1 thymidine kinase and ganciclovir gene therapy as an adjuvant to surgical resection and radiation in adults with previously untreated glioblastoma multiforme. Hum Gene Ther.

[46] Westphal M, Yla-Herttuala S, Martin J, Warnke P, Menei P, Eckland D, Kinley J, Kay R, Ram Z (2013). Adenovirus-mediated gene therapy with sitimagene ceradenovec followed by intravenous ganciclovir for patients with operable high-grade glioma (ASPECT): a randomised, open-label, phase 3 trial. Lancet Oncol.

[47] Harsh GR, Deisboeck TS, Louis DN, Hilton J, Colvin M, Silver JS, Qureshi NH, Kracher J, Finkelstein D, Chiocca EA, Hochberg FH (2000). Thymidine kinase activation of ganciclovir in recurrent malignant gliomas: a gene-marking and neuropathological study. J Neurosurg.

[48] Wakimoto H, Mohapatra G, Kanai R, Curry WT, Yip S, Nitta M, Patel AP, Barnard ZR, Stemmer-Rachamimov AO, Louis DN, Martuza RL, Rabkin SD (2012). Maintenance of primary tumor phenotype and genotype in glioblastoma stem cells. Neuro-Oncol.

[49] Bao S, Wu Q, McLendon RE, Hao Y, Shi Q, Hjelmeland AB, Dewhirst MW, Bigner DD, Rich JN (2006). Glioma stem cells promote radioresistance by preferential activation of the DNA damage response. Nature.

[50] Chan XH, Nama S, Gopal F, Rizk P, Ramasamy S, Sundaram G, Ow GS, Ivshina AV, Tanavde V, Haybaeck J, Kuznetsov V, Sampath P (2012). Targeting glioma stem cells by functional inhibition of a prosurvival oncomiR-138 in malignant gliomas. Cell Rep.

[51] Yang YP, Chien Y, Chiou GY, Cherng JY, Wang ML, Lo WL, Chang YL, Huang PI, Chen YW, Shih YH, Chen MT, Chiou SH (2012). Inhibition of cancer stem cell-like properties and reduced chemoradioresistance of glioblastoma using microRNA145 with cationic polyurethane-short branch PEI. Biomaterials.

[52] Fan X, Khaki L, Zhu TS, Soules ME, Talsma CE, Gul N, Koh C, Zhang J, Li YM, Maciaczyk J, Nikkhah G, DiMeco F, Piccirillo S, Vescovi AL, Eberhart CG (2010). NOTCH pathway blockade depletes CD133-positive glioblastoma cells and inhibits growth of tumor neurospheres and xenografts. Stem Cells.

[53] Jeon JY, An JH, Kim SU, Park HG, Lee MA (2008). Migration of human neural stem cells toward an intracranial glioma. Exp Mol Med.

[54] Auffinger B, Morshed R, Tobias A, Cheng Y, Ahmed AU, Lesniak MS (2013). Drug-loaded nanoparticle systems and adult stem cells: a potential marriage for the treatment of malignant glioma?. Oncotarget.

[55] Cheng Y, Morshed R, Cheng SH, Tobias A, Auffinger B, Wainwright DA, Zhang L, Yunis C, Han Y, Chen CT, Lo LW, Aboody KS, Ahmed AU, Lesniak MC (2013). Nanoparticle-programmed self-destructive neural stem cells for glioblastoma targeting and therapy. Small.

[56] Sasportas LS, Kasmieh R, Wakimoto H, Hingtgen S, van de Water JAJM, Mohapatra G, Figueiredo JL, Martuza RL, Weissleder R, Shah K (2009). Assessment of therapeutic efficacy and fate of engineered human mesenchymal stem cells for cancer therapy. Proc Natl Acad Sci.

[57] Balyasnikova IV, Ferguson SD, Sengupta S, Han Y, Lesniak MS (2010). Mesenchymal stem cells modified with a single-chain antibody against EGFRvIII successfully inhibit the growth of human xenograft malignant glioma. PLoS One.

[58] Wollmann G, Ozduman K, van den Pol AN (2012). Oncolytic virus therapy for glioblastoma multiforme: concepts and candidates. Cancer J.

[59] Forsy P, Roldan G, George D, Wallace C, Palmer CA, Morris D, Cairncross G, Matthews MV, Markert J, Gillespie Y, Coffey M, Thompson B, Hamilton M (2008). A phase I trial of intratumoral administration of reovirus in patients with histologically confirmed recurrent malignant gliomas. Mol Ther.

[60] Harrow S, Papanastassiou V, Harland J, Mabbs R, Petty R, Fraser M, Hadley D, Patterson J, Brown SM, Rampling R (2004). HSV1716 injection into the brain adjacent to tumour following surgical resection of high-grade glioma: safety data and long-term survival. Gene Ther.

[61] Markert JM, Medlock MD, Rabkin SD, Gillespie GY, Todo T, Hunter WD, Palmer CA, Feigenbaum F, Tornatore C, Tufaro F, Martuza RL (2000). Conditionally replicating herpes simplex virus mutant, G207 for the treatment of malignant glioma: results of a phase I trial. Gene Ther.

[62] Cheema TA, Wakimoto H, Fecci PE, Ning J, Kuroda T, Jeyaretna DS, Martuza RL, Rabkin SD (2013). Multifaceted oncolytic virus therapy for glioblastoma in an immunocompetent cancer stem cell model. Proc Natl Acad Sci.

[63] Ogi C, Aruga A (2013). Immunological monitoring of anticancer vaccines in clinical trials. Oncoimmunol.

[64] Chang CN, Huang YC, Yang DM, Kikuta K, Wei KJ, Kubota T, Yang WK (2011). A phase I/II clinical trial investigating the adverse and therapeutic effects of a postoperative autologous dendritic cell tumor vaccine in patients with malignant glioma. J Clin Neurosci.

[65] Jie X, Hua L, Jiang W, Feng F, Feng G, Hua Z (2012). Clinical application of a dendritic cell vaccine raised against heat-shocked glioblastoma. Cell Biochem Biophys.

[66] Phuphanich S, Wheeler C, Rudnick J, Mazer M, Wang H, Nuno M, Richardson J, Fan X, Ji J, Chu R, Bender JG, Hawkins ES, Chirag G, Black KL, Yu JS (2013). Phase I trial of a multi-epitope-pulsed dendritic cell vaccine for patients with newly diagnosed glioblastoma. Cancer Immunol Immunother.

[67] Muragaki Y, Maruyama T, Iseki H, Tanaka M, Shinohara C, Takakura K, Tsuboi K, Yamamoto T, Matsumura A, Matsutani M, Karasawa K, Shimada K, Yamaguchi N, Nakazato Y, Sato K, Uemae Y, Ohno T, Okada Y, Hori T (2011). Phase I/IIa trial of autologous formalin-fixed tumor vaccine concomitant with fractionated radiotherapy for newly diagnosed glioblastoma. J Neurosurg.

[68] Sampson JH, Heimberger AB, Archer GE, Aldape KD, Friedman AH, Friedman HS, Gilbert MR, Herndon JE, McLendon RE, Mitchell DA, Reardon DA, Sawaya R, Schmittling RJ, Shi W, Vredenburgh JJ, Bigner DD (2010). Immunologic escape after prolonged progression-free survival with epidermal growth factor receptor variant III peptide vaccination in patients with newly diagnosed glioblastoma. J Clin Oncol.

[69] Ji J, Judkowski VA, Liu G, Wang H, Bunying A, Li Z, Xu M, Bender J, Pinilla C, Yu JS (2014). Identification of novel human leukocyte antigen-a*0201-restricted, cytotoxic T lymphocyte epitopes on CD133 for cancer stem cell immunotherapy. Stem Cells Transl Med.

[70] Billingham RE, Brent L, Medawar PB (1954). Quantitative studies on tissue transplantation immunity. II. The origin, strength and duration of actively and adoptively acquired immunity. Proc R Soc Lond B Biol Sci.

[71] Eshhar Z, Waks T, Gross G, Schindler DG (1993). Specific activation and targeting of cytotoxic lymphocytes through chimeric single chains consisting of antibody-binding domains and the gamma or zeta subunits of the immunoglobulin and T-cell receptors. Proc Natl Acad Sci.

[72] Miao H, Choi BD, Suryadevara CM, Sanchez-Perez L, Yang S, De Leon G, Sayour EJ, McLendon R, Herndon JE, Healy P, Archer GE, Bigner DD, Johnson LA, Sampson JH (2014). EGFRvIII-specific Chimeric antigen receptor T cells migrate to and kill tumor deposits infiltrating the brain parenchyma in an invasive xenograft model of glioblastoma. PLoS One.

[73] Imai C, Mihara K, Andreansky M, Nicholson IC, Pui CH, Geiger TL, Campana D (2004). Chimeric receptors with 4-1BB signaling capacity provoke potent cytotoxicity against acute lymphoblastic leukemia. Leukemia.

[74] Carpenito C, Milone MC, Hassan R, Simonet JC, Lakhal M, Suhoski MM, Varela-Rohena A, Haines KM, Heitjan DF, Albelda SM, Carroll RG, Riley JL, Pastan I, June CH (2009). Control of large, established tumor xenografts with genetically retargeted human T cells containing CD28 and CD137 domains. Proc Natl Acad Sci.

[75] Zhao Y, Wang QJ, Yang S, Kochenderfer JN, Zheng Z, Zhong X, Sadelain M, Eshhar Z, Rosenberg SA, Morgan RA (2009). A Herceptin-based Chimeric antigen receptor with modified signaling domains leads to enhanced survival of transduced T lymphocytes and antitumor activity. J Immunol.

[76] Brentjens R, Yeh R, Bernal Y, Riviere I, Sadelain M (2010). Treatment of chronic lymphocytic leukemia with genetically targeted autologous T cells: case report of an unforeseen adverse event in a phase I clinical trial. Mol Ther.

[77] Kochenderfer JN, Dudley ME, Feldman SA, Wilson WH, Spaner DE, Maric I, Stetler-Stevenson M, Phan GQ, Hughes MS, Sherry RM, Yang JC, Kammula US, Devillier L, Carpenter R, Nathan DA, Morgan RA, Laurencot C, Rosenberg SA (2012). B-cell depletion and remissions of malignancy along with cytokine-associated toxicity in a clinical trial of anti-CD19 chimeric-antigen-receptor-transduced T cells. Blood.

[78] Grupp SA, Kalos M, Barrett D, Aplenc R, Porter DL, Rheingold SR, Teachey DT, Chew A, Hauck B, Wright JF, Milone MC, Levine BL, June CH (2013). Chimeric antigen receptor-modified T cells for acute lymphoid leukemia. N Engl J Med.

[79] Le Huu D, Matsushita T, Jin G, Hamaguchi Y, Hasegawa M, Takehara K, Fujimoto M (2012). IL-6 blockade attenuates the development of murine sclerodermatous chronic graft-versus-host disease. J Invest Dermatol.

[80] Pule MA, Savoldo B, Myers GD, Rossig C, Russell HV, Dotti G, Huls MH, Liu E, Gee AP, Mei Z, Yvon E, Weiss HL, Liu H, Rooney CM, Heslop HE, Brenner MK (2008). Virus-specific T cells engineered to coexpress tumor-specific receptors: persistence and antitumor activity in individuals with neuroblastoma. Nat Med.

[81] Barkholt L, Flory E, Jekerle V, Lucas-Samuel S, Ahnert P, Bisset L, Buscher D, Fibbe W, Foussat A, Kwa M, Lantz O, Maciulaitis R, Palomaki T, Schneider CK, Sensebe L, Tachdjian G, Tarte K, Tosca L, Salmikangas P (2013). Risk of tumorigenicity in mesenchymal stromal cell-based therapies–bridging scientific observations and regulatory viewpoints. Cytotherapy.

